# Clay mineral composition of upland soils and its implication for pedogenesis and soil taxonomy in subtropical China

**DOI:** 10.1038/s41598-021-89049-y

**Published:** 2021-05-06

**Authors:** Ningxiang Ouyang, Yangzhu Zhang, Hao Sheng, Qing Zhou, Yunxiang Huang, Zhan Yu

**Affiliations:** College of Resources and Environment, Hunan Agricultural University, Changsha, 410128 China

**Keywords:** Geology, Mineralogy

## Abstract

Clay minerals are intermediate products generated during soil development, and their neoformation and transformation are closely related to pedogenesis. Here we aimed at identifying the difference in the clay mineral composition of upland soils derived from different parent materials and different soil-forming environments and exploring the importance of clay mineral composition in pedogenesis and soil taxonomy. We sampled 60 soil B horizons in Hunan Province of subtropical China by digging soils derived from granite (GR), slate and shale (SS), Quaternary red clay (QRC), limestone (LS), and sandstone (SDS). The clay mineral composition and its correlation with parent materials, elevation, micro-topography, and pedogenic processes were investigated using X-ray diffraction and Pearson’s correlation analysis. The clay mineral was dominated by kaolinite, followed by 2:1-type minerals (illite and vermiculite), and a small fraction of mixed-layer minerals. The composition of soil clay minerals varied with parent materials. Kaolinite was predominant in soils derived from GR and LS; mixed-layer minerals prevailed in QRC, whereas illite and vermiculite were prevalent in SDS. In addition, elevation and micro-climate could also explain the variations in clay mineral composition. Increase in elevation was associated with decreased 1:1 clay mineral content and increased 2:1 clay mineral content, especially in soils developed from LS. The composition and content of clay minerals indicated that Ferrosols, Ultisols, and Acrisols had undergone intense weathering; Primosols, Entisols, and Leptosols were characterised by weak weathering, and Plinthic Ali-Udic Cambosols, Plinthudults, and Plinthosols were characterised by strong redox status. This study suggests that clay mineral composition is related to the parent material, climate, and micro-topography, and that it can serve as an indicator of pedogenesis and soil type in subtropical China.

## Introduction

Clay minerals are among the essential components of the solid soil phase, and their composition and relative content can affect many physical and chemical properties and the nutrient status of the soil^1^. The composition and relative content of clay minerals depend on the parent material, climate, and micro-topography^2,3^. In addition, clay mineral composition differs depending on the degree of pedogenesis^4^.

Clay minerals are formed through the neoformation and transformation of primary minerals derived from the weathering product of the parent material^5^. The mineral content and texture of parent materials can influence the formation of clay minerals^6,7^; therefore, the composition and characteristics of clay minerals are closely related to those of the parent materials. The pedogenic environment can also substantially influence the transformation of clay minerals through temperature and precipitation^2^. 1:1-type kaolinite is the dominant clay mineral in subtropical and tropical areas with high air temperature^3,8–10^. In contrast, 2:1-type clay minerals are readily detected in temperate and cool-temperate zones^11,12^. Clay mineral types constantly change as the soil weathers and develops. He et al.^13^ reported that young soils showed an initial illite-formation stage, and that the content of 2:1-type clay minerals decreased over time in the soil chronosequence. Several studies have indicated that there are significant differences in clay mineral composition and content among different soil genetic horizons and soil types^14,15^. Therefore, studies on the effects of different parent materials and pedogenic environments on the composition and content of clay minerals and their dynamic variation among different soil types are of theoretical importance. Several studies have focused on the influence of a single environmental factor, such as parent material or climate type, on the composition of clay minerals^8–10,16^. In some studies, authors have used only one classification system, such as the World Reference Base for Soil Resources (WRB)^17^, Soil Taxonomy (ST)^18^, or Chinese Soil Taxonomy (CST)^19^, to examine the variation in clay mineral composition among different soil types^20–23^. A few studies have investigated the variation in the composition of clay minerals derived from diverse parent materials (more than three parent materials) and the effects of comprehensive environmental factors (e.g., elevation and micro-topography) on clay minerals in the subtropics. However, the dynamic changes in clay minerals among different soil types based on multiple soil classification systems (CST, ST, and WRB) have received limited attention.

Owing to its diversity of parent materials, complex topography, and changeable climate, the subtropical region of China has diverse soil types^24,25^. Desilication and ferrallitisation are common in climates with high air temperatures and precipitation, and this results in the formation of a LAC-ferric horizon (lower-activity clay and free iron oxide rich) in Ferrosols. Clay migration is active in soils characterised by strong eluviation, and it occurs in soils originating from coarse parent materials. These soils easily develop an argic horizon (clay illuviation), which is necessary for Argosols formation. In areas with low air temperature, parent materials tend to be weakly weathered. The soils derived from these parent materials can form a cambic horizon (slight siallitisation), which is necessary for Cambosols formation. On steep slopes, Primosols are common due to the dynamic balance between soil formation and erosion^25,26^.

Hunan Province is home to an ideal series of soils that enable investigation of the effects of parent materials (more precisely, five parent materials) and topography (plains, hills, and mountains) on the composition of clay minerals under warm and humid subtropical climate. The parent rocks or materials in the upland soils of the Hunan Province mainly consist of Quaternary red clay (QRC), granite (GR), slate and shale (SS), sandstone (SDS), and limestone (LS)^27^. Thus, the pedogenic processes of upland soils vary widely because of the diversity of parent materials and the complex topography. It has been reported that the variation in clay mineral composition of Stagnic Anthrosols (paddy soil) is a consequence of diverse parent materials present in Hunan Province^23^; however, there have only been a few studies on the upland soils (natural soils) in this area. Therefore, in the present study, we selected 60 upland soils derived from five parent materials in the Hunan Province and analysed the effect of different parent materials, soil environments, diagnostic horizons, and soil types on clay mineral types and contents using X-ray diffraction. The goals of this study were to (i) investigate the effects of diverse parent materials and environmental factors (elevation and micro-topography) on soil clay mineral composition and (ii) explore the evolution of clay minerals during soil development and its implications for soil taxonomy.

## Materials and methods

### Study area and field sampling

**Figure 1:**
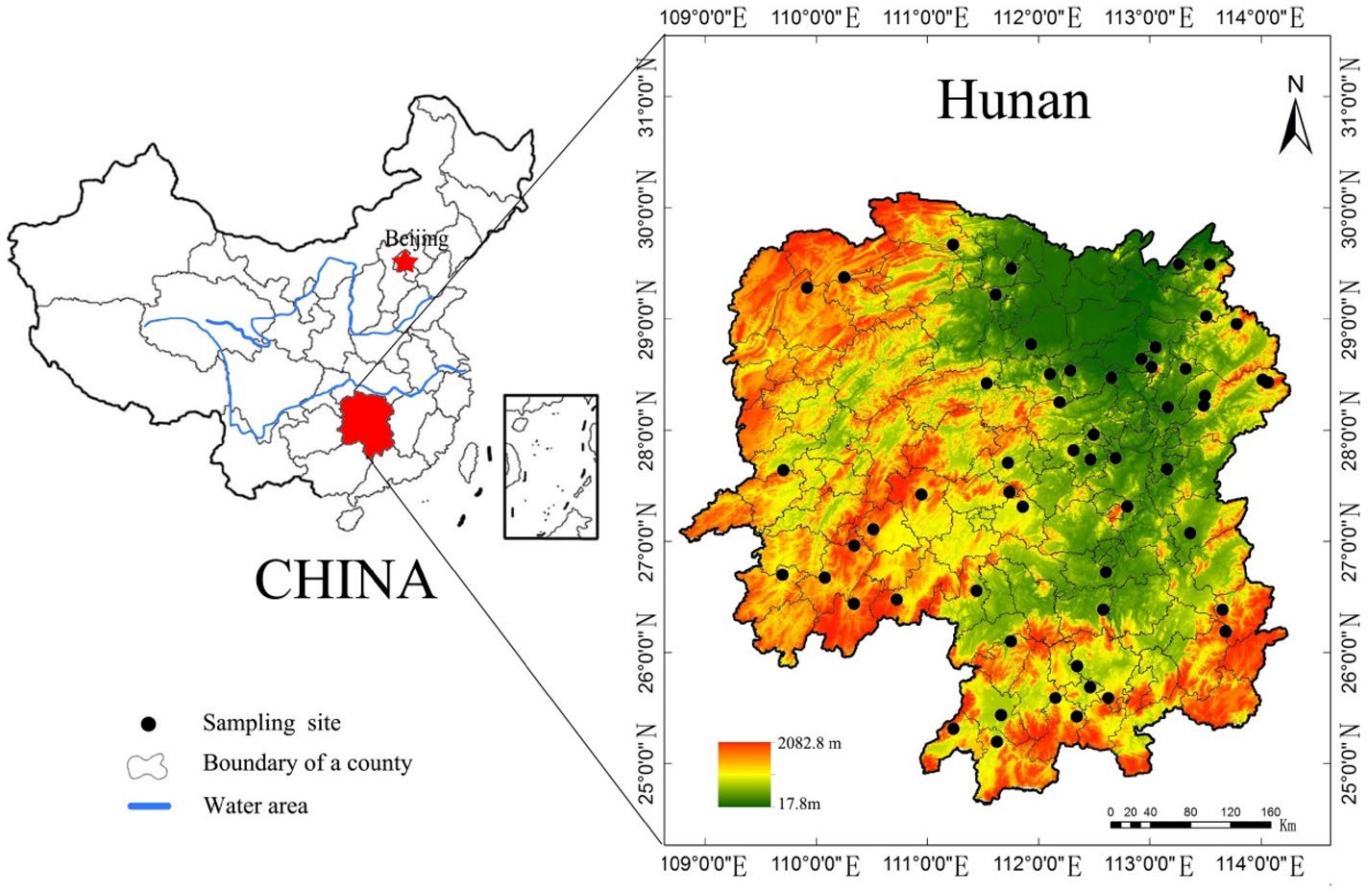
Study area and sampling sites.

This study was conducted in Hunan Province, south-central China (Fig. 1). Hunan Province is located in the middle reaches of the Yangtze River, bordering Dongtinghu Lake in the north. It covers a land area of 211,800 km^2^, characterised by the presence of hills and basins in the centre, mountains in the east, south, and west, and plains in the north. Hunan has a mid-subtropical, seasonally humid climate characterised by moderate air temperature, abundant precipitation, and clear differentiation between wet and dry seasons. The annual sunshine duration is 1300–1800 h, and the annual average air temperature is 16–18 °C. The area is frost-free 260–310 days a year, and the annual precipitation ranges from 1200 to 1700 mm^27^. Sixty sites were selected, covering elevations from 41 to 650 m a.s.l. (Table 1). Parent materials of the soils in the Hunan province mainly included granite (GR), slate and shale (SS), Quaternary red clay (QRC), limestone (LS), and sandstone (SDS). The formation period of GR consists of three geological times, namely the Caledonian, Indosinian, and Yanshanian ages. The primary minerals in GR include feldspar, quartz, mica, and amphibole. SS are formed from moderately metamorphic slate and sedimentary shale. The main slate rocks are argillaceous slate, siliceous slate, and silt slate. Shale is mainly composed of carbonaceous shale, silt shale, and sandy shale. The LS in this region is mainly composed of marine sedimentary carbonate rocks, and its stratigraphic chronology mainly runs from the Devonian to the Permian period. With respect to minerals, LS is mainly composed of calcite and often contains mixed penetrations (sand, clay, dolomite, and silica). Soils developed from QRC, which is formed from glaciofluvial deposits dissolved in the Quaternary interglacial period of the Cenozoic era, are usually sticky due to intense mineral weathering and heavy material illuviation. The SDS in the area mostly consists of marine sedimentary clastic rocks and mainly includes siltstone, sandstone (quartz sandstone, feldspar quartz sandstone, and argillaceous sandstone), and conglomerate. SDS contained minerals, including quartz sand, feldspar sand, and iron siliceous cement^27^.

**Table 1:** Fundamental information about study sites and soil classification.

Profile	Horizon	Depth (cm)	Elevation (m)	Landform†	Parent material	Chinese soil taxonomy (soil subgroup)	Soil taxonomy (soil subgroup)	World reference base for soil resources (second level)
CS16	Bw	50–90	126	LH-MS	Slate and shale (SS)	Typic Argi-Udic Ferrosols	Typic Paleudults	Xanthic Acrisols (Dystric, Loamic)
XT04	Bt	30–60	107	LH-MS		Typic Argi-Udic Ferrosols	Typic Paleudults	Dystric Chromic Sideralic Cambisols (Loamic, Ochric)
YIY02	AC	20–60	107	LH-BS		Lithic Usti-Orthic Primosols	Lithic Ustorthents	Dystric Lithic Leptosols (Loamic, Ochric)
YZ01	Bt	27–70	209	HH-MS		Xanthic Ali-Udic Argosols	Typic Paleudults	Xanthic Alisols (Clayic, Hyperdystric, Ochric)
YY08	Bw	45–105	48	LH-MS		Typic Hapli-Udic Ferrosols	Oxic Dystrudepts	Hyperdystric Xanthic Sideralic Cambisols (Loamic, Ochric)
HH10	Bt	30–65	231	HH-MS		Alic Acidi-Udic Argosols	Typic Paleudults	Xanthic Alisols (Cutanic, Loamic)
ZZ03	Bw	20–50	87	LH-LS		Xanthic Ali-Udic Argosols	Typic Paleudults	Xanthic Alisols (Cutanic, Differentic, Hyperdystric, Loamic)
YIY07	BC	85–115	59	LH-MS		Typic Ali-Udic Cambosols	Typic Humudepts	Cambic Umbrisols (Chromic,Clayic,Dystric)
YY09	Bw	30–80	103	LH-LS		Typic Ali-Udic Cambosols	Typic Dystrudepts	Hyperdystric Chromic Cambisols (Loamic, Ochric)
ZZ06	Bw	25–65	190	LS-MS		Typic Ali-Udic Cambosols	Typic Dystrudepts	Hyperdystric Xanthic Cambisols (Loamic, Ochric)
YIY01	Bw	56–97	226	LH-BS		Typic Ferri-Udic Cambosols	Typic Dystrudepts	Eutric Xanthic Cambisols (Loamic, Ochric)
CZ02	BC	50–130	606	LM-MS		Typic Ferri-Udic Cambosols	Typic Dystrudepts	Dystric Xanthic Cambisols (Loamic, Ochric)
CS02	Bt	62–137	63	LH-MS	Quaternary red clays (QRC)	Typic Argi-Udic Ferrosols	Typic Paleudults	Rhodic Ferric Acrisols (Clayic, Cutanic, Hyperdystric)
CS11	Bls	33–71	41	LH-MS		Typic Ali-Udic Argosols	Typic Plinthudults	Aric Plinthosols (Clayic, Dystric, Ochric)
CS15	Bw	40–90	109	LH-LS		Rhodic Hapli-Udic Ferrosols	Oxic Dystrudepts	Dystric Rhodic Sideralic Cambisols (Loamic, Ochric)
ZZ02	Bt	40–65	75	LH-US		Typic Ali-Udic Argosols	Typic Paleudults	Chromic Ferric Alisols (Clayic, Cutanic, Hyperdystric)
ZZ08	Bt	25–95	105	LH-LS		Typic Argi-Udic Ferrosols	Typic Paleudults	Rhodic Acrisols (Clayic, Cutanic, Hyperdystric)
XT03	Bt	60–150	77	LH-LS		Typic Ali-Udic Argosols	Typic Paleudults	Chromic Alisols (Clayic, Cutanic, Hyperdystric)
YY04	Bw	20–60	69	LH-US		Plinthic Ali-Udic Cambosols	Typic Dystrudepts	Plinthofractic Plinthosols (Clayic, Dystric, Ochric)
YY06	Bw	55–95	56	LH-LS		Mottlic Ali-Udic Cambosols	Typic Dystrudepts	Dystric Chromic Cambisols (Clayic, Ferric, Ochric)
YY07	Bls	60–120	83	LH-MS		Plinthic Ali-Udic Cambosols	Typic Plinthudults	Alic Umbric Plinthosols (Clayic, Hyperdystric)
CD01	Bw	55–120	58	LH-US		Typic Ali-Udic Cambosols	Typic Dystrudepts	Hyperdystric Chromic Cambisols (Clayic, Ochric)
CD06	Bw	40–80	66	LH-MS		Typic Ali-Udic Cambosols	Typic Humudepts	Cambic Umbrisols (Chromic, Clayic, Dystric)
HY05	Bw	40–110	107	LH-LS		Plinthic Argi-Udic Ferrosols	Plinthic Paleudults	Rhodic Plinthic Acrisols (Cutanic, Differentic, Hyperdystric)
LY03	Bt	60–120	179	LM-MS	Granite (GR)	Typic Argi-Udic Ferrosols	Typic Paleudults	Chromic Acrisols (Cutanic, Hyperdystric)
LY04	Bt	49–107	482	LM-LS		Typic Ali-Udic Argosols	Typic Paleudults	Chromic Alisols (Cutanic, Dystric, Loamic)
LY21	Bw	11–51	650	LM-MS		Xanthic Ali-Udic Cambosols	Typic Dystrudepts	Hyperdystric Xanthic Cambisols (Loamic, Ochric)
XT02	Bt	55–100	89	LH-LS		Typic Argi-Udic Ferrosols	Typic Paleudults	Chromic Acrisols (Cutanic, Dystric, Loamic)
YY01	Bw	40–90	287	LH-MS		Typic Ali-Udic Cambosols	Typic Dystrudepts	Hyperdystric Xanthic Cambisols (Loamic, Ochric)
YY05	Bt	47–70	87	LH-LS		Typic Argi-Udic Ferrosols	Typic Paleudults	Acric Umbrisols (Chromic, Clayic, Hyperdystric, Sideralic)
YY10	Bw	75–100	128	LH-MS		Typic Hapli-Udic Ferrosols	Oxic Dystrudepts	Sideralic Chromic Cambisols (Dystric, Loamic)
YIY04	Bw	40–120	68	LH-MS		Rhodic Hapli-Udic Ferrosols	Oxic Dystrudepts	Cambic Umbrisols (Chromic, Clayic, Hyperdystric, Sideralic)
CZ06	Bw	25–75	350	HH-MS		Red Ferri-Udic Cambosols	Typic Dystrudepts	Dystric Cambisols (Loamic, Ochric)
SY08	Bw	60–120	416	HH-LS		Xanthic Hapli-Udic Ferrosols	Oxic Dystrudepts	Hyperdystric Xanthic Sideralic Cambisols (Loamic, Ochric)
HY07	Bt	25–48	81	LH-LS		Typic Hapli-Udic Ferrosols	Oxic Dystrudepts	Hyperdystric Xanthic Sideralic Cambisols (Loamic, Ochric)
CS18	Bt	30–60	100	LH-LS		Typic Argi-Udic Ferrosols	Typic Paleudults	Hyperdystric Chromic Sideralic Cambisols (Loamic, Ochric)
ZZ05	Bt	26–60	263	LH-MS	Limestone (LS)	Typic Argi-Udic Ferrosols	Typic Paleudults	Acric Umbrisols (Clayic, Hyperdystric, Sideralic)
YZ03	Bt	90–140	201	LH-US		Typic Argi-Udic Ferrosols	Typic Paleudults	Acric Umbrisols (Clayic, Hyperdystric, Rhodic, Sideralic)
YZ05	Bts	60–100	228	LH-MS		Mottlic Argi-Udic Ferrosols	Ultic Hapludalfs	Chromic Ferric Lixisols (Clayic, Cutanic, Differentic, Hypereutric)
YZ06	Bts	80–135	290	HH-MS		Mottlic Argi-Udic Ferrosols	Aquic Paleudults	Xanthic Ferric Lixisols (Clayic, Cutanic, Differentic, Hypereutric)
YZ08	Bts	60–130	380	HH-MS		Trunic Argi-Udic Ferrosols	Typic Paleudults	Chromic Ferric Acrisols (Clayic, Cutanic, Differentic, Hyperdystric)
CZ04	Bt	75–110	404	HH-MS		Humic Ferri-Udic Argosols	Ultic Hapludalfs	Luvic Umbrisols (Clayic, Hypereutric, Rhodic)
CZ05	Bt	100–160	255	HH-MS		Typic Argi-Udic Ferrosols	Typic Paleudults	Acric Umbrisols (Clayic, Dystric, Rhodic, Sideralic)
SY04	Bt	20–43	462	LM-LS		Red Ferri-Udic Argosols	Typic Rhodudalfs	Placic Rhodic Ferric Luvisols (Clayic, Cutanic, Differentic, Hypereutric)
SY07	Bs	80–130	343	LH-MS		Mottlic Hapli-Udic Ferrosols	Oxic Dystrudepts	Dystric Xanthic Sideralic Cambisols (Clayic, Ferric, Ochric)
SY09	Bt	50–100	320	LH-US		Xanthic Argi-Udic Ferrosols	Typic Paleudults	Chromic Ferric Acrisols (Clayic, Cutanic, Differentic)
LD01	Bt	30–65	174	LH-TS		Typic Argi-Udic Ferrosols	Typic Paleudults	Chromic Acrisols (Clayic, Cutanic, Differentic, Hyperdystric)
HY02	Bt	50–105	92	LH-MS		Trunic Argi-Udic Ferrosols	Typic Paleudults	Chromic Ferric Acrisols (Clayic, Cutanic, Differentic)
XT05	BC	40–110	59	LH-MS	Sandstone (SDS)	Typic Ali-Udic Cambosols	Typic Dystrudepts	Dystric Chromic Cambisols (Loamic, Ochric)
YIY03	Bw	80–120	147	LH-US		Typic Ali-Udic Cambosols	Typic Dystrudepts	Hyperdystric Chromic Cambisols (Loamic, Ochric)
CD02	Bt	65–110	197	LH-MS		Typic Ferri-Udic Argosols	Typic Paleudults	Alic Umbrisols (Eutric, Loamic)
YZ02	Bw	35–75	238	LH-US		Typic Ali-Udic Cambosols	Typic Dystrudepts	Hyperdystric Chromic Cambisols (Loamic, Ochric)
YZ04	Bw	15–60	216	LH-US		Typic Ali-Udic Cambosols	Typic Dystrudepts	Hyperdystric Xanthic Cambisols (Loamic, Ochric)
YZ07	Bw	25–95	309	HH-MS		Typic Ali-Udic Cambosols	Typic Dystrudepts	Hyperdystric Xanthic Cambisols (Loamic, Ochric)
SY03	Bw	45–110	317	LH-MS		Typic Hapli-Udic Ferrosols	Oxic Dystrudepts	Cambic Umbrisols (Hyperdystric, Loamic, Sideralic)
SY06	Bw	12–48	375	LH-MS		Typic Ali-Udic Cambosols	Typic Dystrudepts	Hyperdystric Xanthic Cambisols (Loamic, Ochric)
SY10	Bt	80–160	370	LH-MS		Humic Ali-Udic Argosols	Typic Paleudults	Xanthic Alisols (Cutanic, Differentic, Hyperdystric, Loamic)
SY01	Bw	20–50	379	HH-MS		Humic Ali-Udic Cambosols	Typic Humudepts	Cambic Umbrisols (Clayic, Hyperdystric)
XX03	Bt	20–60	421	HH-MS		Xanthic Ali-Udic Argosols	Typic Paleudults	Xanthic Alisols (Clayic, Cutanic, Differentic)
ZJJ05	Bt	100–165	484	HH-MS		Humic Ferri-Udic Argosols	Typic Paleudults	Xanthic Luvisols (Cutanic, Differentic, Hypereutric, Loamic)

†LH-TS: Low Hill-Top of slope; LH-US: Low Hill-Upslope; LH-MS: Low Hill-Mesoslope; LH-LS:Low Hill-Lower Slope; LH-BS: Low Hill-Bottom slopes; HH-US: High Hill-Upslope; HH-MS: High Hill-Mesoslope; HH-LS: High Hill-Lower Slope; HH-BS: High Hill-Bottom slopes; LM-MS: Low Mountain-Mesoslope; LM-LS: Low Mountain-Lower Slope.

We referred to *The Manual of Soil Description and Sampling* for collecting and describing the soil samples^28^. The soils were classified as Ferrosols, Argosols, Cambosols, or Primosols using the CST classification system; as Ultisols, Alfisols, Inceptisols, and Entisols using the ST; and as Acrisols, Lixisols, Alisols, Luvisols, Plinthosols, Umbrisols, Cambisols, and Leptosols according to WRB^17–19^. These soil classifications are consistent with those of previous studies that investigated correlations among the CST, ST, and WRB systems^25,29^. To avoid human interference and fully reflect the nature of the soil and the inheritance of parent materials, only the weathering B horizons in the soil profiles were sampled.

### Soil mineralogy

The collected soil samples (clay fraction < 2 μm) were characterised using an X-ray diffractometer (Model D/Max–rA; Rigaku, Tokyo, Japan) with the following parameters: radiation, Ni-filtered CuKα; voltage, 40 kV; current, 40 mA. The diaphragm system was set as follows: divergence slit (DS) = anti-scatter slit (SS) = 1° and receiving slit (RS) = 0.3 mm. It measured from 3° to 30° 2θ at a scan rate of 2° 2θ min^−1^ and a step size of 0.02° 2θ. For preparing samples for orientation sampling, the clay fraction samples were air-dried (AD), saturated with ethylene–glycol (EG) at 70 °C for 3 h, and heated (HT) to 450 °C or 600 °C for 2.5 h. The X-ray patterns were analysed using XPowder, a software package for powder X-ray diffraction analysis. Kaolinite was identified based on the presence of 0.72 and 0.358 nm peaks after being AD and saturated with EG, and the 0.72 nm peak disappeared after heating to 600 °C. Then, illite was identified based on the presence of a 1.00 nm peak after being AD and saturated with EG, and the peak persisted after heating. Vermiculite and chlorite were distinguished by heat treatment. Shrinkage of the 1.42 nm peak to 1.00–1.03 nm after heating indicated the presence of vermiculite. I/S was identified based on the presence of a 1.45–1.54 nm peak, which slightly expanded after saturation with EG and shifted to 1.00 nm after heating. I/V was determined based on an unaltered D1 zone at 1.00–1.42 nm and appeared in the D3 zone at 0.50–0.47 nm; in the D2 zone, it shifted to < 1.0 nm after heating. The relative content of clay minerals was calculated based on the height of the diffraction peak^30–35^.

### Soil physicochemical properties

Soil total K content was determined after digesting the samples with solid NaOH in a silver crucible at 450 °C for 15 min, and then gradually increasing the temperature. The concentration of K was determined using a flame photometer. Soil texture was determined using the pipette method and classified using the United States Department of Agriculture (USDA) classification standards. Soil organic carbon (SOC) was measured using the K_2_Cr_2_O_7_ wet oxidation method. Soil pH was measured using a glass electrode placed in a solution of soil and distilled water (1:2.5). The cation exchange capacity (CEC) and exchangeable Ca, Mg, K, and Na were determined using 1 M NH_4_OAc at pH 7. In each sample, the Fe_d_ was extracted using sodium dithionite-citrate. The total concentration of Fe, Al, and Si was determined by treating samples with Li_2_CO_3_-H_3_BO_3_ after heating at 900 °C for 0.5 h, and then, the solution was analysed by inductively coupled plasma atomic emission spectrometry (ICP-AES) (Table 2)^36^. CEC/clay was calculated using the following equation^19^:

**Table 2:** Physicochemical properties of upland soils derived from different parent materials in Hunan Province, China.

Soil properties†	SS n = 12	QRC n = 12	GR n = 12	LS n = 12	SDS n = 12
pH (H_2_O)	4.7 ± 0.4 b^‡^	4.8 ± 0.2 b	4.8 ± 0.3 b	5.5 ± 0.5 a	4.8 ± 0.4 b
SOC, g kg^−1^	4.5 ± 2.5 b	4.2 ± 1.2 b	4.0 ± 1.5 b	5.1 ± 2.3 b	7.6 ± 4.6 a
Sand, g kg^−1^	348 ± 169 b	214 ± 138 c	458 ± 154 a	68 ± 50 d	228 ± 127 c
Silt, g kg^−1^	346 ± 126 ab	313 ± 107 bc	229 ± 51 c	283 ± 122 bc	412 ± 95 a
Clay, g kg^−1^	306 ± 93 c	474 ± 72 b	313 ± 131 c	650 ± 152 a	360 ± 95 c
Al_2_O_3_, g kg^−1^	158 ± 37 b	148 ± 17 b	201 ± 30 a	209 ± 54 a	139 ± 41 b
SiO_2_, g kg^−1^	661 ± 96 ab	666 ± 62 ab	604 ± 75 bc	538 ± 92 c	709 ± 81 a
K_2_O, g kg^−1^	21.8 ± 8.3 b	18.1 ± 5.2 b	32 ± 10.4 a	17 ± 6 b	17.8 ± 7.2 b
Fe_2_O_3_, g kg^−1^	61.4 ± 24.9 b	59 ± 11.7 b	50.8 ± 16.2 b	87 ± 28.6 a	48.2 ± 15.4 b
Fe_d_, g kg^−1^	25.4 ± 6.7 b	28.5 ± 6.6 b	20 ± 9.6 b	45.6 ± 15.9 a	26.3 ± 6.9 b
CEC, cmol kg^−1^	10.9 ± 4.8 b	15 ± 2.2 a	13.2 ± 3.4 ab	16.8 ± 5.8 a	13.5 ± 5.2 ab
CEC/clay, cmol kg^−1^ clay	34 ± 9.3 a	27.6 ± 5.8 a	30.6 ± 17 a	23.3 ± 5.4 a	36 ± 10.5 a

†SOC: soil organic carbon; CEC: cation exchange capacity; CEC/clay, cation exchange capacity of the clay fraction; Fed: dithionite-citrate-bicarbonate (DCB)-extractable.

^‡^Mean ± standard deviation. Means followed by different letters indicate the data were statistically different at P < 0.05 among different parent materials.

$$\mathrm{CEC}/\mathrm{clay } \; \left({\text{cmol} \; \text{kg}}^{-1} \; \mathrm{ clay}\right)=\frac{\mathrm{CEC} \, (\mathrm{by }\;\; \text{NH}_{4}\,\mathrm{OAC }\,{pH}_{7}) \, ({\mathrm{cmol} \; \text{kg}}^{-1} \; \mathrm{ soil})}{\mathrm{clay} \, ({\mathrm{g} \; \text{kg}}^{-1})}\times 1000$$

### Statistical analysis

The data were processed and analysed using SPSS 22.0 (IBM, Chicago, IL, USA). Before performing one-way ANOVA, the physicochemical properties of soils derived from different parent materials were tested for normal distribution and variance homogeneity using Kolmogorov–Smirnov and Levene test, respectively. Pearson’s correlation coefficient was used to test the correlation between clay minerals and the physical and chemical properties of the soil. The least significant difference test was used to test for significance. Significant differences are denoted by *P < 0.05, and extremely significant differences are indicated with **P < 0.01. All figures were created using Origin 9.1 software.

## Results

### Composition and relative content of clay minerals in different parent materials

**Figure 2:**
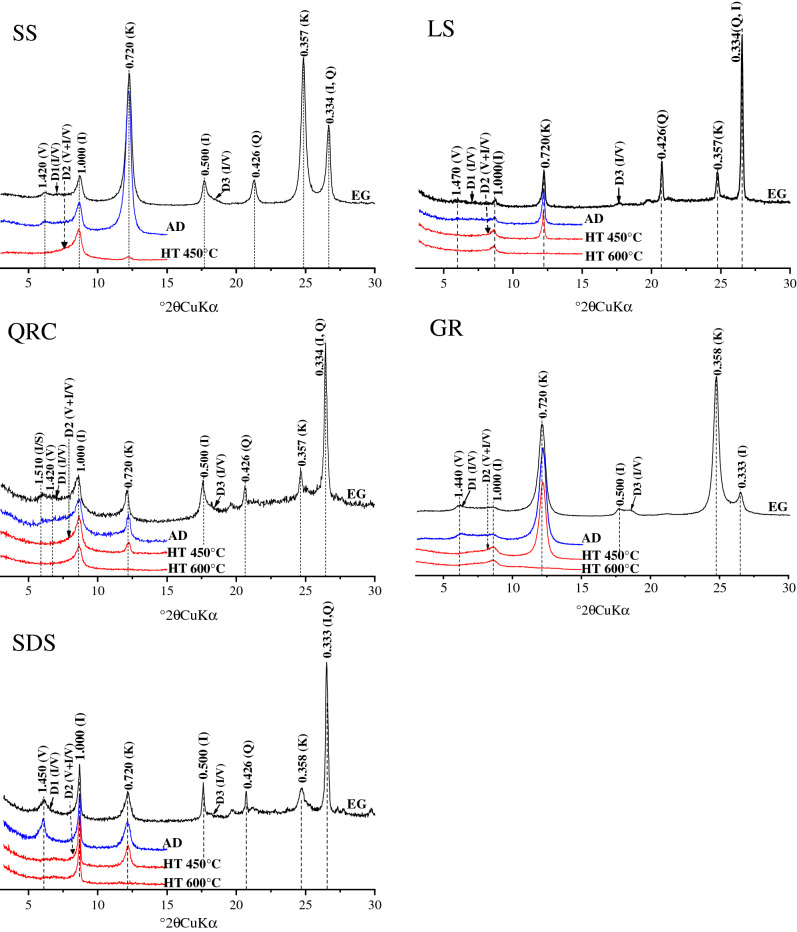
X-ray diffraction patterns of the representative upland soil profiles derived from different parent materials (K: kaolinite; I: illite; V: vermiculite; I/S: illite/smectite mixed-layer mineral; I/V: illite/vermiculite mixed-layer mineral; Q: quartz; AD: air-dried sample; EG: ethylene–glycol saturated sample; HT 450 °C: sample heated at 450 °C; HT 600 °C: sample heated at 600 °C; SS: slate and shale; LS: limestone; QRC: Quaternary red clay; GR: granite; SDS: sandstone).

The clay minerals in upland soils were composed of 1:1-type clay minerals kaolinite (0.71–0.73 nm, 0.35–0.36 nm), 2:1-type clay minerals illite (1.0–1.03 nm), vermiculite (1.42–1.49 nm shifting to 1.0–1.03 nm after heating), and mixed-layer minerals illite/vermiculite (I/V) (determined by the unchanged D1 zone at 1.00–1.42 nm, appeared in the D3 zone at 0.50–0.47 nm and shifted to < 1.0 nm in the D2 zone after heating), and illite/smectite (I/S) (1.45–1.66 nm shifted to 1.0–1.02 nm after heating) (Fig. 2). The I/S mixed-layer minerals were only revealed in the plinthic horizon derived from QRC.

**Figure 3:**
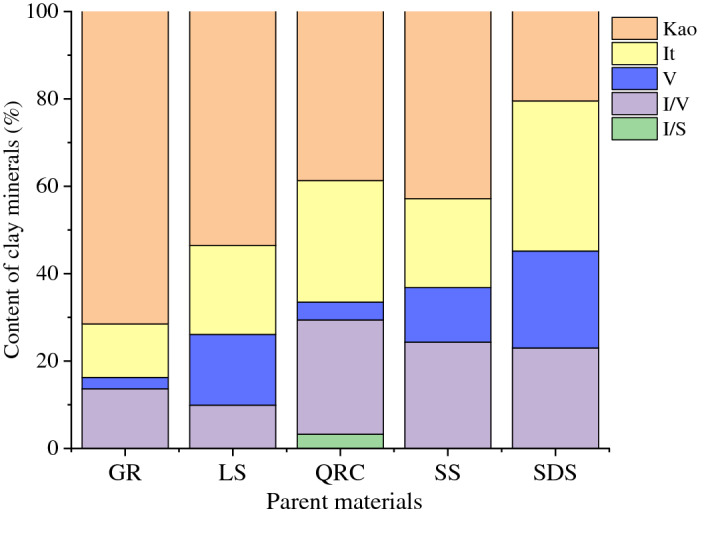
Relative content of clay minerals of studied upland soils derived from different parent materials (Kao: kaolinite; It: illite; V: vermiculite; I/S: illite/smectite mixed-layer mineral; I/V: illite/vermiculite mixed-layer mineral; GR: granite; LS: limestone; QRC: Quaternary red clay; SS: slate and shale; SDS: sandstone).

The relative content of clay minerals varied among upland soils derived from different parent materials (Fig. 3). In GR, the relative content of kaolinite was the highest (72%), and the content of illite and vermiculite were the lowest (12% and 3%, respectively). The kaolinite content (54%) in LS was slightly lower than that in GR, whereas the 2:1-type illite (20%) and vermiculite (16%) content in LS were higher than those in GR. The upland soils in LS contained a small amount of I/V mixed-layer minerals (10%). Kaolinite (39%), illite (28%), and I/V mixed-layer minerals (26%) were predominant in QRC, whereas vermiculite was less dominant (4%). In SS, the kaolinite and illite content varied substantially. The kaolinite content (43%) in SS was higher than that in QRC, but the content of illite (20%) and mixed-layer minerals (24%) was lower. In SS, the relative content of vermiculite (13%) was lower than that of other clay minerals. In SDS, the content of 1:1-type kaolinite (21%) was the lowest, and the content of 2:1-type illite (34%) and vermiculite (22%) were the highest. The content of I/V mixed-layer minerals (23%) in SDS was relatively higher than that in GR and LS.

### Differences in clay mineral composition and relative content among diverse pedogenic environments

**Figure 4:**
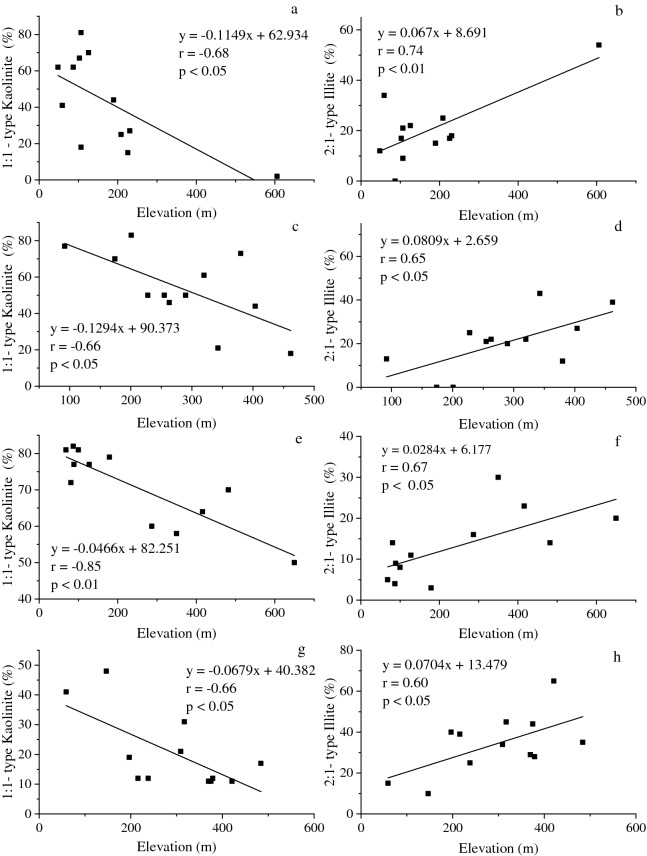
Relationships between relative content of clay minerals and elevation for the studied soils (**a**,**b**: slate and shale; **c**,**d**: limestone; **e**,**f**: granite; **g**,**h**: sandstone).

Correlation analysis revealed that clay mineral composition was correlated with elevation (Fig. 4). For QRC, the range of elevation was small, and the elevation difference was less than 70 m; therefore, no correlation analysis was conducted. Apart from QRC, the kaolinite content in other parent materials was positively correlated with elevation (SS: r = − 0.68, LS: r = − 0.66, GR: r = − 0.85, SDS: r = − 0.66), but an opposite trend was observed for 2:1-type illite (SS: r = 0.74, LS: r = 0.65, GR: r = 0.67, SDS: r = 0.60). Nevertheless, the magnitude of variation at distinct elevations differed among the parent materials. The change in the slope of clay minerals in LS (k_Kao_ = − 0.13, k_2:1-type_ = 0.08) was higher than that in the other three parent materials. This indicated that with increasing elevation, the kaolinite content decreased, whereas the 2:1-type illite content increased. Compared to the clay minerals of other parent materials, the clay minerals of LS were more sensitive to variations in elevation.

**Figure 5:**
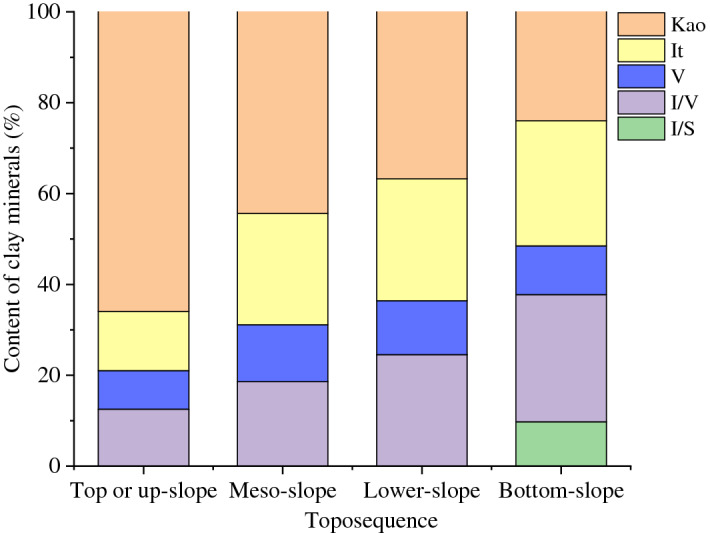
Relationships between relative contents of clay minerals and toposequence for the studied soils (Kao: kaolinite; It: illite; V: vermiculite; I/S: illite/smectite mixed-layer mineral; I/V: illite/vermiculite mixed-layer mineral).

In addition to the correlation between elevation and the composition and relative content of clay minerals, we also observed a correlation between topographic position and clay minerals (Fig. 5). By comparing the topographic position with the composition and content of clay minerals, we found that the samples with high quantities of mixed-layer minerals and 2:1-type clay minerals were mostly collected from the slope foot or toe, whereas soil samples with a high 1:1-type kaolinite content were mostly collected from the slope shoulder or crest.

### Differences in the composition and relative content of clay minerals at different diagnostic horizons and soil types (i.e., soil orders)

**Figure 6:**
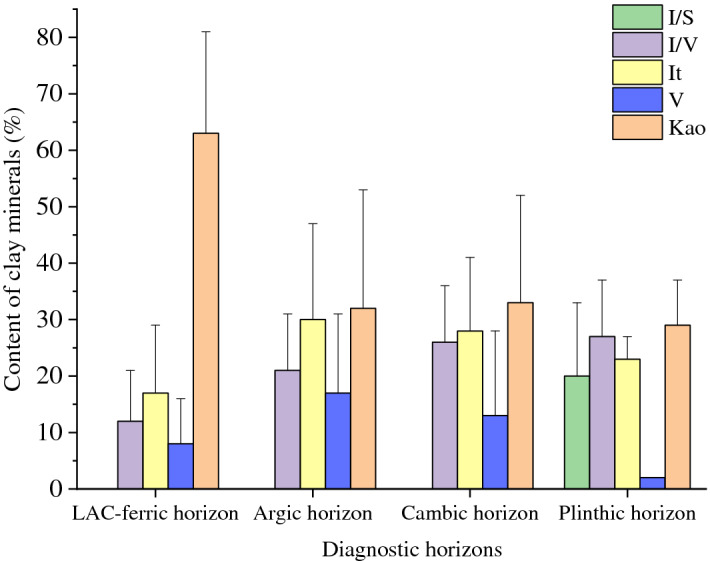
Relative content of clay minerals of different diagnostic horizons (Kao: kaolinite; It: illite; V: vermiculite; I/S: illite/smectite mixed-layer mineral; I/V: illite/vermiculite mixed-layer mineral).

**Figure 7:**
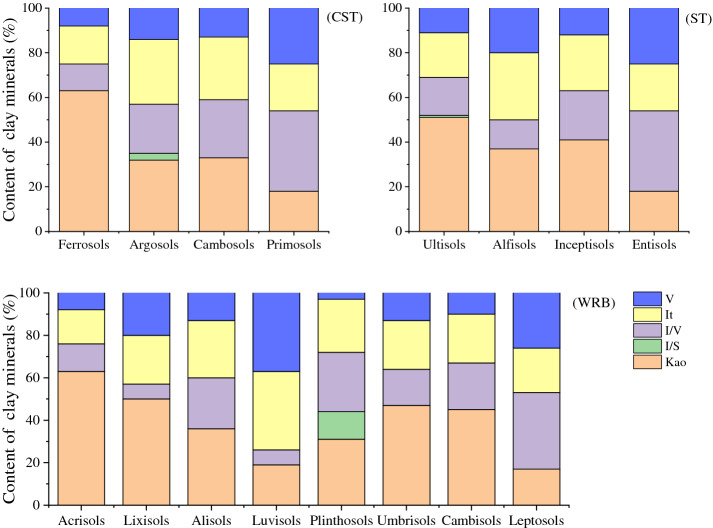
Relative contents of clay minerals of different soil types (i.e., soil orders) (Kao: kaolinite; It: illite; V: vermiculite; I/S: illite/smectite mixed-layer mineral; I/V: illite/vermiculite mixed-layer mineral; CST: Chinese Soil Taxonomy; ST: Soil Taxonomy; WRB: World Reference Base for Soil Resources).

We identified 60 soil profiles with four diagnostic horizons based on the CST, i.e., LAC-ferric horizon, argic horizon, cambic horizon, and plinthic horizon^19^. The 60 soil profiles were classified as Ferrosols, Argosols, Cambosols, and Primosols in CST^19^; as Ultisols, Alfisols, Inceptisols, and Entisols in ST^18^; and as Acrisols, Lixisols, Alisols, Luvisols, Plinthosols, Umbrisols, Cambisols, and Leptosols in WRB^17^. The clay mineral composition was significantly different among the diagnostic horizons and soil types (i.e., soil orders) (Figs. 6, 7). The dominant clay mineral in the LAC-ferric horizon was 1:1-type kaolinite (63%), followed by illite (17%), I/V mixed-layer minerals (12%), and a small amount of vermiculite (8%). In the plinthic horizon, the relative content of mixed-layer minerals, including I/V (27%) and I/S (20%) mixed-layer minerals, was the highest (47%). The plinthic horizon also contained a high percentage of kaolinite (29%) and illite (23%) and a small amount of vermiculite (2%). The differences in clay mineral composition were negligible in the argic and cambic horizons.

Among all the soil types (i.e., soil orders), the content of 1:1-type kaolinite was the highest in Ferrosols (63%), followed by 2:1 clay minerals (I + V: 25%) and I/V mixed-layer minerals (12%). In Primosols, the content of 2:1-type clay mineral (I + V: 46%) and I/V mixed-layer mineral (36%) were the highest, and the content of 1:1-type clay mineral kaolinite (18%) was the lowest. The variation in clay mineral composition in Argosols and Cambosols was negligible. However, the content of mixed-layer minerals (I/V + I/S) was the highest in Plinthic Ali-Udic Cambosols (40%). Similar results were obtained using the ST and WRB classification systems. The content of 1:1-type kaolinite was the highest in Ultisols (51%) and Acrisols (63%), and the lowest in Entisols (18%) and Leptosols (17%). Mixed-layer minerals (I/V + I/S) were predominant in Plinthudults (47%) and Plinthosols (41%). This indicated that the clay mineral composition and relative content have a strong effect on Ferrosols, Ultisols, and Acrisols (high development degree); Primosols, Entisols, and Leptosols (low development degree); and Plinthic Ali-Udic Cambosols, Plinthudults, and Plinthosols (strong redox status).

### Correlation between clay minerals and soil properties

**Figure 8:**
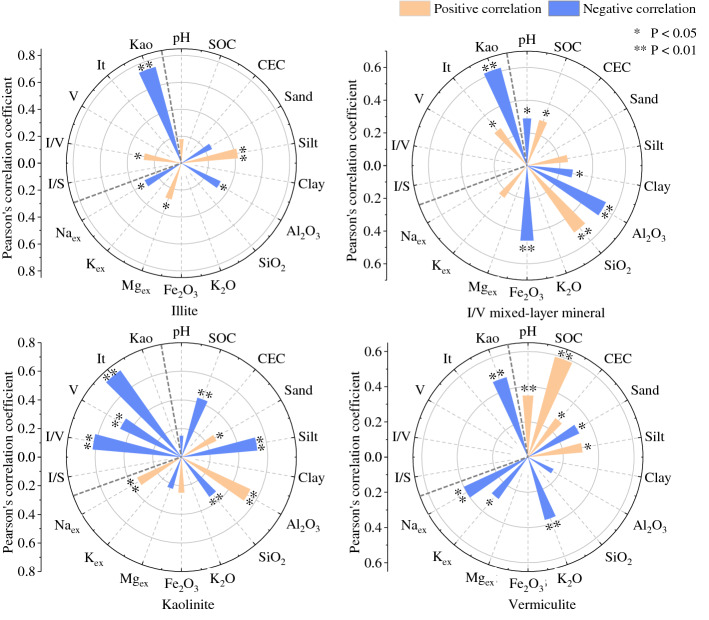
Correlativity of the clay minerals with some physico-chemical properties in studied soils.

Correlations between the composition and relative content of clay minerals with soil properties—such as SOC, CEC, soil texture, and pH—were determined (Fig. 8). Pearson’s correlation analysis showed that the content of 1:1-type kaolinite was positively correlated with the sand content (r = 0.27, P < 0.05), significantly and positively correlated with aluminium oxide and exchangeable Na (r_Al_ = 0.53, P < 0.01; r_Na_ = 0.34, P < 0.01), and significantly and negatively correlated with silt content, SOC, and silicon oxide (r_silt_ = − 0.53, P < 0.01; r_SOC_ = − 0.43, P < 0.01; r_Si_ = − 0.34, P < 0.01). The content of 2:1-type illite was negatively correlated with aluminium oxide and exchangeable Na (r_Al_ = − 0.32, P < 0.05; r_Na_ = − 0.30, P < 0.05) and significantly and positively correlated with silt content (r = 0.42, P < 0.01). Vermiculite content was positively correlated with SOC and pH (r_SOC_ = 0.59, P < 0.01; r_pH_ = 0.35, P < 0.01) and negatively correlated with potassium oxide and exchangeable Na (r_K_ = − 0.37, P < 0.01; r_Na_ = − 0.40, P < 0.01). The I/V mixed-layer mineral content was positively correlated with silicon oxide (r = 0.50, P < 0.01) and negatively correlated with aluminium oxide and iron oxide (r_Al_ = − 0.53, P < 0.01; r_Fe_ = − 0.46, P < 0.01). Additionally, 1:1-type kaolinite was significantly negatively correlated with 2:1-type illite, vermiculite, and I/V mixed-layer minerals (r_I_ = − 0.74, P < 0.01; r_V_ = − 0.47, P < 0.01; r_I/V_ = − 0.62, P < 0.01). Illite showed a positive relationship with I/V mixed-layer minerals (r = 0.28, P < 0.05).

Pearson’s correlations between the clay minerals and CEC/clay and Fe_d_ are shown in Table 3. Kaolinite was negatively correlated with CEC/clay and positively and significantly correlated with Fe_d_, whereas 2:1-type clay minerals (illite and vermiculite) were negatively correlated with CEC/clay and positively correlated with Fe_d_.

**Table 3:** Correlation coefficients between the clay minerals and CEC/clay and Fe_d_.

Parent material	Clay minerals	CEC/clay (cmolc kg^−1^ clay)		Fe_d_ (g kg^−1^)	
		R	P	R	P
SS n = 12	Kaolinite	− 0.638*	0.026	0.626*	0.030
	2:1-type	0.614*	0.034	− 0.369	0.238
QRC n = 12	Kaolinite	− 0.627*	0.029	0.642*	0.024
	2:1-type	0.370	0.236	− 0.370	0.236
GR n = 12	Kaolinite	− 0.580*	0.048	0.593*	0.042
	2:1-type	0.596*	0.041	− 0.567	0.055
LS n = 12	Kaolinite	− 0.722**	0.008	0.582*	0.047
	2:1-type	0.767**	0.004	− 0.358	0.253
SDS n = 12	Kaolinite	− 0.684*	0.014	0.604*	0.038
	2:1-type	0.513	0.088	− 0.149	0.644

*Significant at the 0.05 level.

**Significant at the 0.01 level.

## Discussion

### Effects of parent materials on the composition and relative content of clay minerals

The type of parent material and the degree to which it is weathered have a major influence on the neoformation and transformation of clay minerals. These factors can result in variations in clay mineral composition and content in the same area due to differences in the mineral composition and texture of parent materials^7^. GR is mainly composed of feldspar, quartz, biotite, and other primary minerals^27^, and in the present study, the content of sand and K_2_O in GR was significantly higher than that in the other four types of parent materials (Table 2). Under acidic and strong leaching conditions, feldspar intensely weathers and hydrolyses, and kaolinite can be mass produced by neoformation^37^. In addition, under conditions of high K content, vermiculation can be inhibited, and biotite can be oxidised and directly weathered to kaolinite^38,39^. Therefore, the kaolinite content in the GR group was generally high (Fig. 3). QRC is composed of Quaternary fluvioglacial sediments, and during development, it is subjected to both sedimentary-weathering soil-forming processes and the glacial-interglacial cycle^40^. Unstable factors in the sedimentary-weathering soil-forming process, strong cyclic climates, and environments that alternate between dry and wet seasons can promote illite alteration, a phenomenon that involves the transformation from single phase to mixed-layer minerals^16,41,42^. Thus, in the present study, the content of mixed-layer minerals in the QRC group was generally high.

Previous studies have shown that the plinthic horizon of QRC was formed on flat, low-lying terrains in a climate that frequently alternated between dry and wet^25^. Due to frequent fluctuations in groundwater, soil aggregates shrink when dry, a phenomenon that is conducive to the formation of cracks^43^. When the groundwater level rises, the soil is in a reducing state in which Fe^3+^ can be transformed into soluble and mobile Fe^2+^, thus promoting the leaching of Fe oxide from the soil. This process results in the formation of the plinthic horizon, which consists of uniform red soils with white veins and white spots^44,45^. Smectite is generally formed in tropical and subtropical areas with dry and wet seasons, flat terrain, and poor drainage^46^. In wet climates with poor drainage conditions, illite transforms into smectite by absorbing Mg^2+^, but in dry conditions, K is fixed in the layer of smectite and promotes the formation of I/S mixed-layer minerals^5,47^. Therefore, the environment is conducive to the transformation of illite to smectite, and the I/S mixed-layer minerals were found in the plinthic horizon.

In the present study, SDS was mainly composed of quartz sand, feldspar sand, and other iron and siliceous cements. The soils developed from SDS are characterised by high gravel content and low degree of development, and they occur at high altitudes (> 300 m)^27^. Therefore, the contents of 2:1-type clay minerals and mixed-layer minerals were the highest in SDS. LS is mainly composed of marine sedimentary carbonate rocks, and its stratigraphic chronology mainly runs from the Devonian to the Permian period^27^. The soils derived from LS are old and exhibit a high degree of weathering. Therefore, 1:1-type kaolinite was the main clay mineral in the soils derived from LS. The relative content of kaolinite in LS was lower than that in GR because of its high viscosity and weak leaching in LS.

The physical and chemical properties of soils developed from SS differ because the SS in the study area is composed of two kinds of parent rocks, i.e., low-grade metamorphic rock slate and sedimentary shale and as their components are relatively complex^27^. Leaching was strong in soils developed from sandy slate with a low pH and high silt content, and therefore, the content of 1:1-type kaolinite was high in these soils. However, leaching was weak in soils derived from clay shale with a high pH and high clay content, and therefore, the content of 2:1-type clay minerals and mixed-layer minerals was high in these soils^48^.

### Effects of pedogenic environment on the composition and relative content of clay minerals

Warm and humid climatic conditions in subtropical regions promote soil weathering and development, and the eluviation and deposition of materials result in the development of acidic, fine-grained, strong-weathering soils characterised by desilication and ferrallitisation^49,50^. These environmental factors mainly contributed to the development of 1:1-type clay mineral kaolinite, and to a certain extent, 2:1-type illite and vermiculite. The mixed-layer minerals were dominated by I/V (Fig. 3). These results were consistent with those of studies on upland soil clay mineral characteristics in other regions in southern China^8–10,51^.

The regional pedogenic environments, especially the elevation and terrain, influence the transformation of clay minerals through temperature and precipitation or via the changes in hydrologic and thermal conditions. In high-altitude conditions with low temperatures, mica primary minerals can form a large amount of illite through depotassication in the mineral interlayer due to weak weathering^52^. In weak acidic and strong leaching environments, vermiculite can be easily formed when soil K and Mg are lost^53–55^. In high-altitude areas, the low temperatures result in decreased H_4_SiO_4_^0^ and ferrallitisation activity, a phenomenon that can inhibit the conversion of 1:1-type kaolinite^56^. However, in low-altitude areas, transformation of 2:1-type clay minerals to kaolinite is promoted by higher temperatures that also increase ferrallitisation and H_4_SiO_4_^0^ activity^56–58^. The transformation of clay minerals can also be affected by erosion in high-altitude areas. In areas at high altitudes or with steep topography, 1:1-type kaolinite is strongly eroded, whereas 2:1-type clay minerals are continuously created from parent materials^59^. In the present study, the content of 1:1-type kaolinite in soils derived from LS, GR, SS, and SDS decreased with increasing elevation, whereas the content of 2:1-type clay mineral showed an opposite trend (Fig. 4). These results were similar to those observed for low mountains and hills (< 1500 m)^59,60^, but different from those found for subalpine or alpine zones (> 1500 m)^61^. However, the varying gradient of clay mineral content with changing elevation differed in diverse climatic zones, even in the same low mountain/hill area. Based on the findings of previous studies on clay minerals in different climatic regions^60,62^, we established a series of regression models to analyse the influence of elevation on the composition and relative content of clay minerals (Table 4). In the present study, the decline in kaolinite content ($\stackrel{-}{\mathrm{k}}$= − 0.09) with elevation was greater than that recorded in tropical (k = − 0.03) and temperate oceanic regions (k = − 0.04)^60,62^. A possible reason for such a result is the relatively stable environmental and climatic conditions that exist in these two regions, which are responsible for the relatively slow material migration and mineral transformation in the soil.

**Table 4:** Results of the literature survey of correlation between clay mineral (kaolinite) and altitude in different climate areas.

Location	Climate	Parent materials	Regression equation	Slope	r	Reference
Hunan Province, South China	Subtropical	Slate and shale	y = − 0.1149x + 62.934	− 0.115	− 0.68	This study
		Limestone	y = − 0.1294x + 90.373	− 0.129	− 0.66	
		Granite	y = − 0.0466x + 82.251	− 0.047	− 0.85	
		Sandstone	y = − 0.0679x + 40.382	− 0.068	− 0.66	
Cameroon plateaus, West Central Africa	Tropical	Metamorphic rocks and plutonic rocks	y = − 0.0245x + 82.258	− 0.025	− 0.35	Nakao et al.^62^
Marlborough Sounds, South Island, New Zealand	Temperate oceanic	Schistose greywacke	y = − 0.0441x + 48.4	− 0.044	− 0.89	Laffan et al.^60^

Nevertheless, the magnitude of variation of clay minerals content in different elevations varies with the type of parent materials. For LS, its weak alkalinity (pH: 5.5 ± 0.5), high clay content (clay: 650 ± 152 g kg^−1^), and slight weathering of minerals at high altitudes were conducive to the transformation of the primary mineral to illite via damouritisation and sericitisation^52,63^. At low altitudes, the high temperature and old stratigraphic chronology of LS (Devonian to Permian) will facilitate the transformation of 2:1-type clay minerals into 1:1-type kaolinite through neoformation or mineral degradation. Therefore, the sensitivity of clay minerals in LS with the variation of altitude was greater than that of the other parent materials.

Micro-topography can affect the transformation of clay minerals in the subsurface horizon by redistributing soil hydrological conditions in the upland soils of the Hunan Province (Fig. 5). The present study showed that a wide distribution of kaolinite in the slope shoulder or crest soils also indicates strong leaching conditions, whereas a high content of 2:1-type clay minerals (illite, vermiculite) and mixed-layer minerals in the slope foot or toe soils suggests frequent wet–dry cycles and weak leaching conditions. These findings were similar to those obtained by Fang et al.^51^, who reported that kaolinite was most frequently found in divergent-site surface soils, whereas 2:1-type clay minerals mainly existed in convergent-site (water-collecting) surface soils. However, the present study showed that the influence of micro-topographical features on the transformation of soil clay minerals could extend to the subsoil.

### Dynamic changes in clay mineral composition among the different soil types (i.e., soil orders)

**Figure 9:**
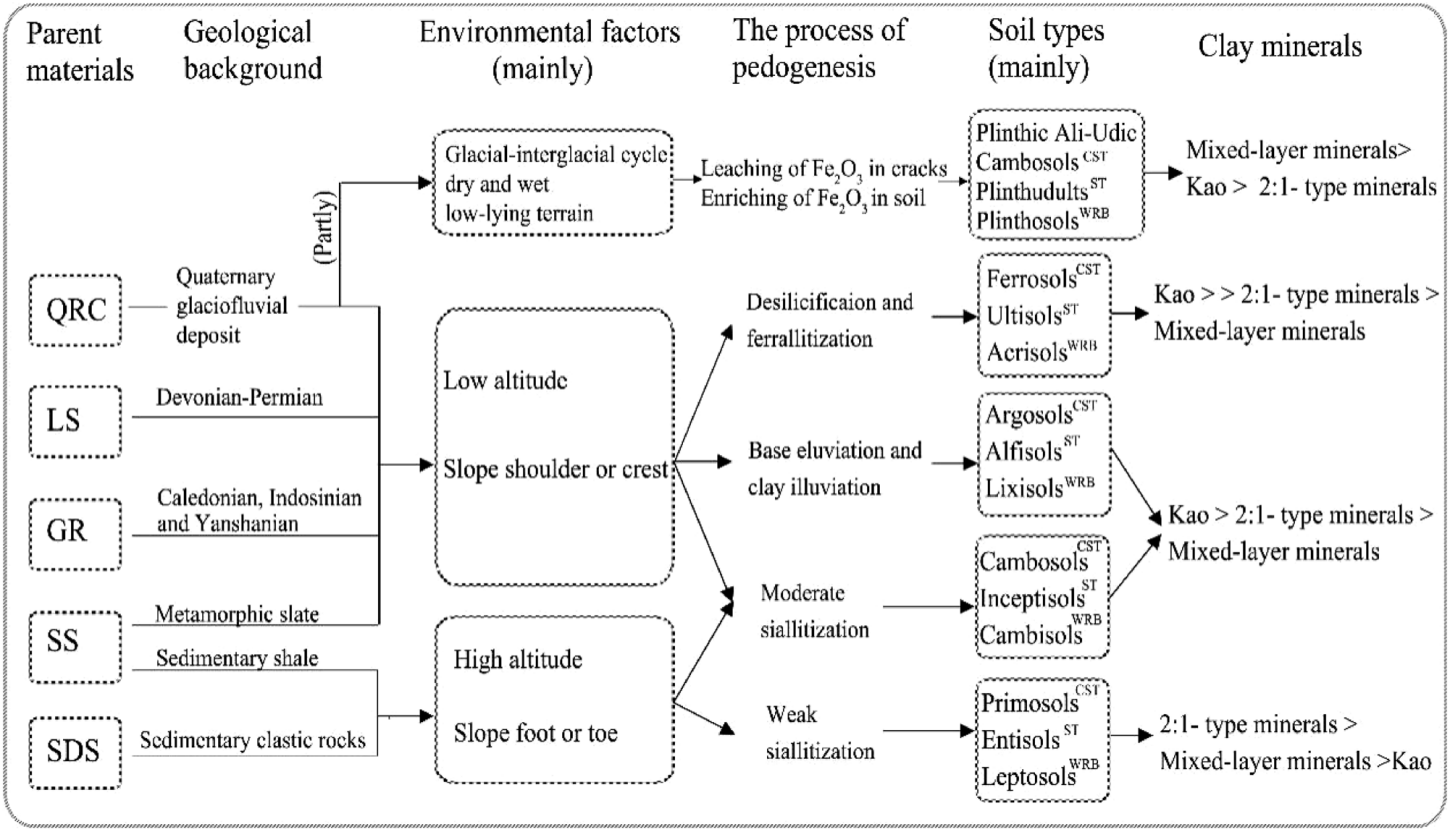
Schematic representation of the conditions leading to evolution of the clay mineral composition in studied upland soils in Hunan, China (GR: granite; LS: limestone; QRC: Quaternary red clay; SS: slate and shale; SDS: sandstone; CST: Chinese Soil Taxonomy; ST: Soil Taxonomy; WRB: World Reference Base for Soil Resources).

Soil classification systems, especially quantitative classification systems, such as the WRB, ST, and CST, are based on the theory of pedogenesis, diagnostic horizons, and diagnostic characteristics. Therefore, different soil types can often reflect different pedogenic processes and development stages^17–19^. Clay minerals develop from primary minerals that have undergone weathering and pedogenesis during soil development. Their composition and relative content can reflect the strength of soil weathering and changes in the soil-forming environment. As the degree of pedogenesis increases (i.e., the CEC/clay content decreases and Fe_d_ content increases), the kaolinite content increases, whereas the content of 2:1 clay minerals decreases (Table 3). In the ST system, 1:1 clay minerals were mostly found in ultisols and oxisols with a high degree of weathering, and 2:1 clay minerals (smectite) were mainly found in the Vertisols that shrink and expand. Conversely, vermiculite and illite were generally found in soil types with less intense weathering, such as Alfisols, Mollisols, and Aridisols^64^. Upon using the WRB, the 1:1-type kaolinite, which has low activity, was identified as the main clay mineral type in Ferralsols, whereas 2:1 clay minerals (such as chlorite, smectite, and vermiculite) were mostly found in Luvisols with a high activity^65^. In the present study, the content of 1:1-type kaolinite was the highest in Ferrosols (CST), Ultisols (ST), and Acrisols (WRB) with moderate ferrallitisation and strong weathering. In contrast, the content of 2:1 clay mineral was the highest in Primosols (CST), Entisols (ST), and Leptosols (WRB) with slight siallitisation and weak weathering, and the content of mixed-layer mineral was the highest in Plinthic Ali-Udic Cambosols (CST), Plinthudults (ST), and Plinthosols (WRB) with a strong redox status (Figs. 6, 9).

## Conclusions

The clay mineral composition at the study sites is representative of the upland soils in Hunan Province. Clay minerals mainly included 1:1 kaolinite, which contains a certain amount of 2:1-type clay minerals such as illite and vermiculite and a small amount of mixed-layer minerals. Our results suggested that the parent material, regional pedogenic environment (elevation and micro-topography), and degree of pedogenesis (soil type) play important roles in the transformation of clay minerals. Kaolinite dominated the mineral distribution patterns in GR (sandy) and LS (old), whereas 2:1-type clay minerals were dominant in SDS (clastic). The content of mixed-layer minerals was high in QRC (glacial-interglacial cycles and wet-dry cycles). In low mountains and hills (< 1500 m), neoformation and transformation of clay minerals were determined by elevation and micro-topography. The influence of elevation on clay mineral transformation in the subtropical monsoon region is greater than that in tropical and temperate oceanic regions. Compared with the parent material, elevation showed a greater effect on the transformation of clay minerals in LS. Micro-topography can modulate the transformation of clay minerals in the subsurface horizon. The composition and relative content of clay minerals can efficiently indicate soil types with a high development degree (Ferrosols, Ultisols, and Acrisols), those with a low development degree (Primosols, Entisols, and Leptosols), and those with a strong redox status (Plinthic Ali-Udic Cambosols, Plinthudults, and Plinthosols).

The factors influencing clay mineral composition are complex and diverse. In the present study, only parent material, climate, and micro-topography were investigated, whereas the effects of vegetation type and pedogenic time were not explored. Future research should focus on these important factors to elucidate the mechanisms underlying clay mineral neoformation and transformation and to assess their significance in pedogenesis.
